# Pain management, prolonged opioid use, initiated anti-rheumatic treatment and psychiatric morbidity in new-onset psoriatic arthritis

**DOI:** 10.1093/rap/rkaf039

**Published:** 2025-04-10

**Authors:** Anna Laine, Paula Muilu, Hannu Kautiainen, Kari Puolakka, Vappu Rantalaiho

**Affiliations:** Department of Medicine, Kanta-Häme Central Hospital, Hämeenlinna, Finland; Centre for Rheumatic Diseases, Tampere University Hospital, Tampere, Finland; Primary Health Care Unit, Kuopio University Hospital, Kuopio, Finland; Folkhälsan Research Center, Helsinki, Finland; Terveystalo Healthcare, Lappeenranta and Turku, Finland; Department of Medicine, Kanta-Häme Central Hospital, Hämeenlinna, Finland; Centre for Rheumatic Diseases, Tampere University Hospital, Tampere, Finland; Faculty of Medicine and Health Technology, Tampere University, Tampere, Finland

**Keywords:** psoriatic arthritis, pain, opioids, psychiatric morbidity

## Abstract

**Objectives:**

To explore the use of DMARDs and painkillers in incident PsA patients.

**Methods:**

From the Finnish Social Insurance Institution register we collected all adult patients granted a special reimbursement (SR) for DMARDs for PsA from 1 January 2010 to 31 December 2014 (*N* = 2678). For each case, three general population controls were matched. The purchases of painkillers, antidepressants, anxiolytics and hypnotics were analysed 1 year before and after the index date (ID; the date the SR was granted) and DMARDs at the ID and 1 year before it.

**Results:**

The year preceding the ID, 51% of the patients purchased any DMARDs, with 31% being MTX. Nevertheless, on the ID the respective percentages increased to 95% and 71%. PsA patients purchased all painkillers significantly more often than their controls before the ID and the purchases peaked at the ID. After that, the purchases of paracetamol and NSAIDs decreased but those of opioids remained at almost the same level. PsA patients purchased antidepressants and hypnotics more often than their controls. The use of the antidepressants, anxiolytics, hypnotics and opioids before the ID was associated with the risk of prolonged opioid use.

**Conclusion:**

A substantial proportion of incident PsA patients are purchasing DMARDs before the ID, which may reflect the difficulty of setting a PsA diagnosis or may represent the treatment of severe skin psoriasis. PsA patients use more painkillers than their matched controls 1 year preceding the diagnosis. Prolonged opioid use is particularly evident among patients using psychiatric medications.

Key messages

- The use of opioids does not decrease as steeply as that of NSAIDs and paracetamol after DMARD initiation in PsA.
- PsA patients use more antidepressants and anxiolytics than their controls and DMARD initiation has no effect on that.
- The use of opioids and psychiatric medications before a PsA diagnosis is a significant risk factor for prolonged opioid use.

## Introduction

PsA is a chronic inflammatory rheumatic disease with a somewhat increasing incidence since the 2000s and affecting men slightly more often than women [1]. Due to the wide spectrum of clinical phenotypes of PsA and lacking biomarkers, setting the diagnosis may be difficult and delayed. Reliable assessment of enthesitis, which is considered a characteristic clinical feature of PsA, is difficult and requires ultrasound imaging findings consistent with clinical findings [2, 3]. Therefore, assessing disease activity can be difficult [4, 5] and therefore PsA patients may have an unmet need for treatment of the inflammatory and pain aspects of the disease [6, 7].

An adequate response is not always achieved with anti-rheumatic medication and differential diagnosis of chronic pain can remain a challenge. Prolonged use of pain medication may serve as a surrogate marker for undertreatment of inflammation in rheumatic disease, but there are other possible explanations such as fibromyalgia, psychiatric comorbidities and addiction to opioids, increasing the risk of prolonged use of painkillers [8–10].

We have earlier shown that patients with incident RA, unspecified arthritis (UA), non-radiographic axial spondylarthritis and AS purchase more painkillers than their controls, with the purchases peaking just before the time of diagnosis and decreasing after that. However, the opioid purchases did not decrease as steeply as purchases of other painkillers, especially among the UA and spondylarthritis patients [11, 12]. In this population-based nationwide trial we wanted to analyse the anti-rheumatic medications initiated in incident PsA patients as well as to compare their use of painkillers and psychiatric medications to matched general population (GP) controls.

## Methods

In Finland, the Pharmaceuticals Pricing Board (PPB) decides which drugs are reimbursed for which indications. Patients with long-term severe diseases such as chronic inflammatory rheumatic diseases, e.g. PsA, can be granted a special reimbursement (SR) of 65% of drug prices. For this, the Social Insurance Institution (SII) of Finland requires a medical certificate from a rheumatologist or a doctor working in a rheumatology clinic that describes the diagnostic procedures and the planned treatment. Since the economic benefit of the SR is significant for the patient, the doctor usually writes the medical certificate the same date the first anti-rheumatic drug is prescribed, generally also being the date of the diagnosis. It takes ≈3–4 weeks for the SII to grant the SR since the certificates are first evaluated by the SII’s insurance physician. The SII maintains a register of the citizens granted SRs, including the patient’s age, sex, date of entitlement and the International Classification of Disease, Tenth Revision (ICD-10) code of the diagnosis that advocates use of the drugs. From this national register we collected all adult patients (≥17 years of age) granted the first SR for anti-rheumatic drugs with the diagnosis of PsA between 1 January 2010 and 31 December 2014. The patients were identified with an ICD-10 code of L40.5. In this study the day of the reimbursement decision was defined as the index date (ID).

To compare the use of painkillers, hypnotics, antidepressants and anxiolytics between PsA patients and the GP, each case was individually matched with three GP controls randomly selected from the Population Register Centre according to age, sex and place of residence on the ID. Controls granted an SR for any inflammatory arthritis before 2010 were excluded.

The Drug Purchase Register (DPR), also maintained by the SII, covers all drug purchases prescribed by physicians and reimbursed on a regular or special level by the National Sickness Insurance Scheme in Finland; information on the amount of the drug and the date of purchase are also included. In the DPR, the drugs are classified according to the Anatomical Therapeutic Chemical (ATC) code developed by the World Health Organization.

The purchases of DMARDs, including both conventional synthetic DMARDs (csDMARDs) and self-injected biologic DMARDs (bDMARDs) and glucocorticoids (GCs), on the ID and 1 year before the ID were evaluated to reflect the possible difficulty of setting the PsA diagnosis.

The purchases of antidepressants (ATC code N06A), anxiolytics (N03AE, N05BA and N06CA01) and hypnotics (N05CD and N05CF) of PsA patients and their controls were collected from the DPR 1 year before and 1 year after the ID. We also analysed the pain medication purchases of PsA patients and their controls 1 year before and 1 year after the ID in 3-month time periods. Purchases of opioids (N02A), NSAIDs (M01A), paracetamol (N02BE01) and gabapentinoids (N03AX12, N03AX16) were collected. Any opioid purchases extending to the period of 4–12 months after the ID were defined as prolonged opioid use. This period was chosen to reflect the use of opioids while on DMARDs: after 3 months of use, response to DMARD therapy should have begun.

The median annual incomes with index increase were collected for PsA patients and their controls from the register maintained by the SII and their education levels from the Population Register Centre (Statistics Finland). Three education levels were reported: basic education level (compulsory basic comprehensive school, classes 1–9), middle level (high school, vocational school and trade school) and high level (including both lower and upper high levels).

### Ethical considerations

Permission to use the databases was obtained from the SII. According to Finnish law, register-based studies do not require the approval of an ethics committee when the study subjects are not contacted.

### Statistical methods

Summary statistics were described using mean and s.d., median and interquartile range (IQR) or number and percentage. The statistical analysis between the cases and the controls was made by generalised linear models with appropriate distribution and link functions. Repeated measures were analysed using generalised estimating equation (GEE) models with a logit link, a binomial distribution and an exchangeable correlation structure. GEE models take into account the correlation between repeated measurements in the same subject; models do not require complete data and can be fit even when individuals do not have observations at all time points. All available data were analysed using the full analysis set (missing data were handled by using available-case analysis). Multivariate and stepwise (forward selection: probability for entry 0.10 and removal 0.05) logistic regression analyses were performed to evaluate factors associated with opioid purchases. To ensure that our model was not overfitting, we internally validated our models using cross-validation. Stata 18.0 (StataCorp, College Station, TX, USA) was used for the statistical analyses.

## Results

Between 2010 and 2014 a total of 2678 PsA patients (≥17 years of age) and 7895 controls were identified. A scant minority (49%) of the study population were women and their mean age was 49 years (s.d. 13).

The purchases of DMARDs and GCs by the patients on the ID as well as 1 year before the ID are presented in Table 1. As expected, the proportion of patients purchasing any DMARD (95%) increased on the ID. Somewhat surprisingly, half of the patients had DMARD purchases 1 year before the ID. The most used csDMARD was MTX, followed by SSZ, whereas the share of other csDMARDs was small. Only a small proportion of patients started a self-injected bDMARD on the ID. GCs were purchased at least once by a quarter of the patients.

**Table 1.** DMARD and GC purchasers among the 2678 PsA patients the year before and on the ID

Purchase	Year before the ID, *n* (%)	On the ID, *n* (%)
Any DMARD	1374 (51)	2537 (95)
Conventional DMARDs		
Methotrexate	817 (31)	1899 (71)
Sulfasalazine	378 (14)	695 (26)
Leflunomide	40 (1)	79 (3)
Ciclosporin	32 (1)	34 (1)
Azathioprine	1 (0)	1 (0)
Other conventional DMARDs^a^	75 (3)	59 (2)
Self-injected bDMARDs	35 (1)	119 (4)
GCs	638 (24)	635 (24)

a Including hydroxychloroquine, auranofin and aurathiomalate.

The controls were slightly more educated than the PsA patients: of the PsA patients, 21% had basic, 61% middle and 18% high levels of education, whereas the proportions for controls were 21%, 57% and 22%, respectively (*P* = 0.002). There were no differences in annual incomes between the cases and the controls [annual median income with index increase €29 846 (IQR 16 629–43 033) *vs* €30 009 (IQR 18 237–41 138); *P* = 0.89].

Antidepressants and hypnotics were purchased more often by PsA patients than their controls. The initiation of anti-rheumatic medication on the ID did not lower the proportion of PsA patients purchasing antidepressants, anxiolytics and hypnotics at the 1-year follow-up (Table 2).

**Table 2.** PsA patients and their controls purchasing antidepressants, anxiolytics and hypnotics during the year before and the year after the ID

Purchase	PsA patients (*N* = 2678), *n* (%)	Controls (*N* = 7895), *n* (%)	RR (95% CI)
Antidepressants			
Before	342 (13)	772 (10)	1.31 (1.16, 1.47)
After	351 (13)	773 (10)	1.34 (1.19, 1.51)
Anxiolytics			
Before	144 (5)	357 (5)	1.18 (0.98, 1.43)
After	143 (5)	354 (4)	1.19 (0.99, 1.45)
Hypnotics			
Before	255 (10)	491 (6)	1.53 (1.32, 1.77)
After	242 (9)	514 (7)	1.39 (1.20, 1.61)
Anxiolytics or hypnotics			
Before	346 (13)	750 (9)	1.36 (1.21, 1.53)
After	335 (13)	768 (10)	1.29 (1.14, 1.45)

The age- and sex-adjusted risk ratios (RRs) for drug purchases with 95% confidence intervals (CIs) are shown.

**Figure 1.**
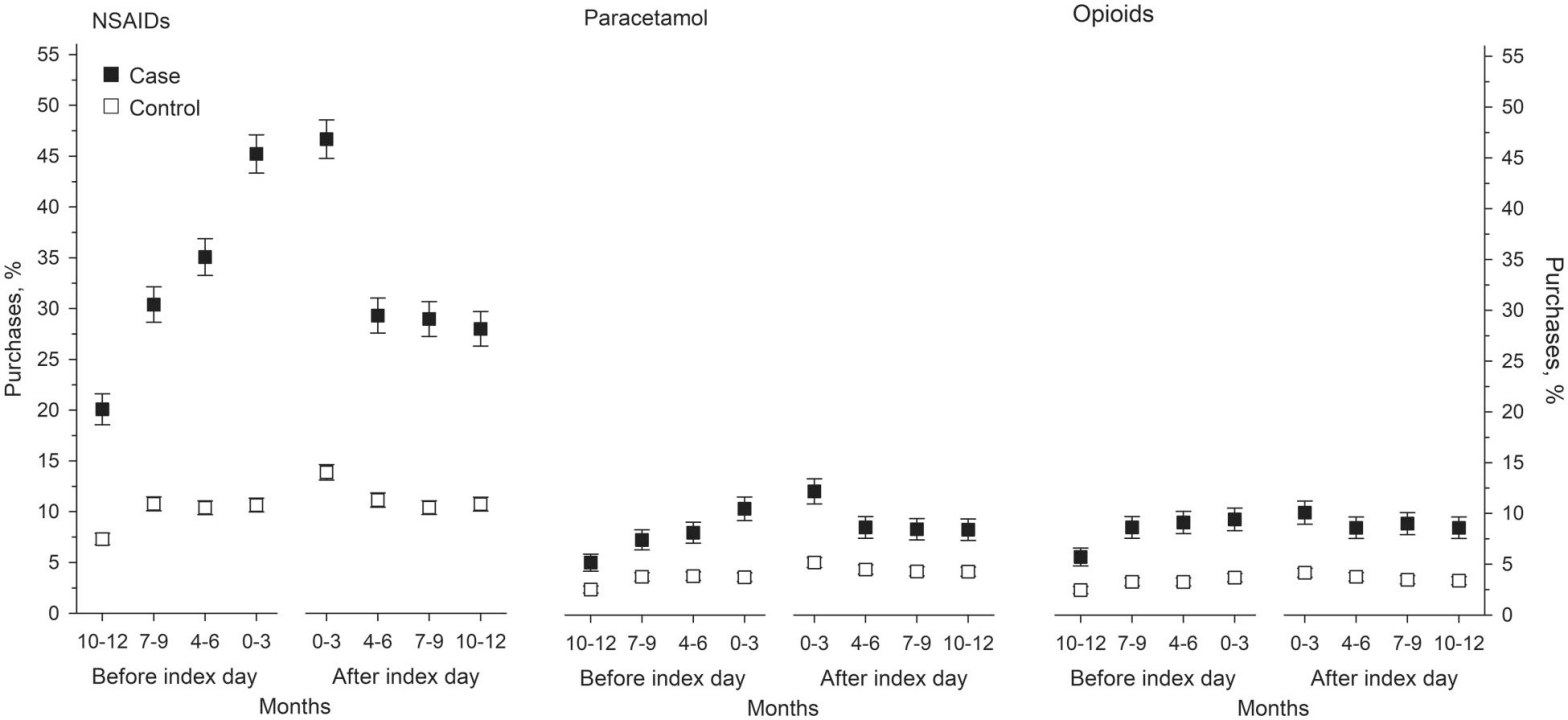
The proportion (%) of NSAID, paracetamol and opioid purchasers among PsA patients and their controls 1 year before and after the ID, divided into 3-month periods

The proportion of paracetamol, NSAID and opioid purchasers among PsA patients was higher than among the controls during the year before and the year after the ID (Fig. 1). PsA patients’ NSAID and paracetamol purchases peaked during the last 3-month period before the ID and clearly decreased after the ID, whereas opioid purchases stayed relatively high during the whole 2-year observation time (Fig. 1). Only a small proportion (1–2%) of patients and controls purchased gabapentinoids, and there were no differences between the groups.

**Figure 2.**
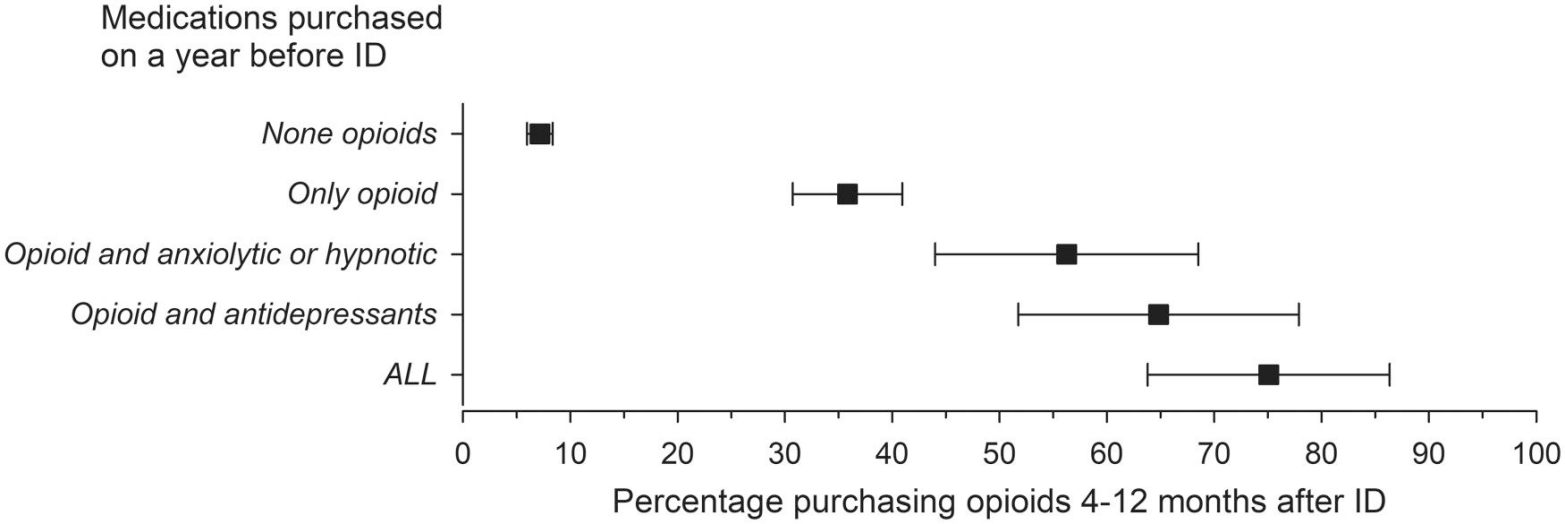
The cumulative additional effect of opioid, anxiolytic and antidepressant purchases by PsA patients during 1 year before the ID on prolonged opioid use. The results are adjusted for sex and age. Prolonged opioid use is defined as opioid purchases 4–12 months after the ID

**Figure 3.**
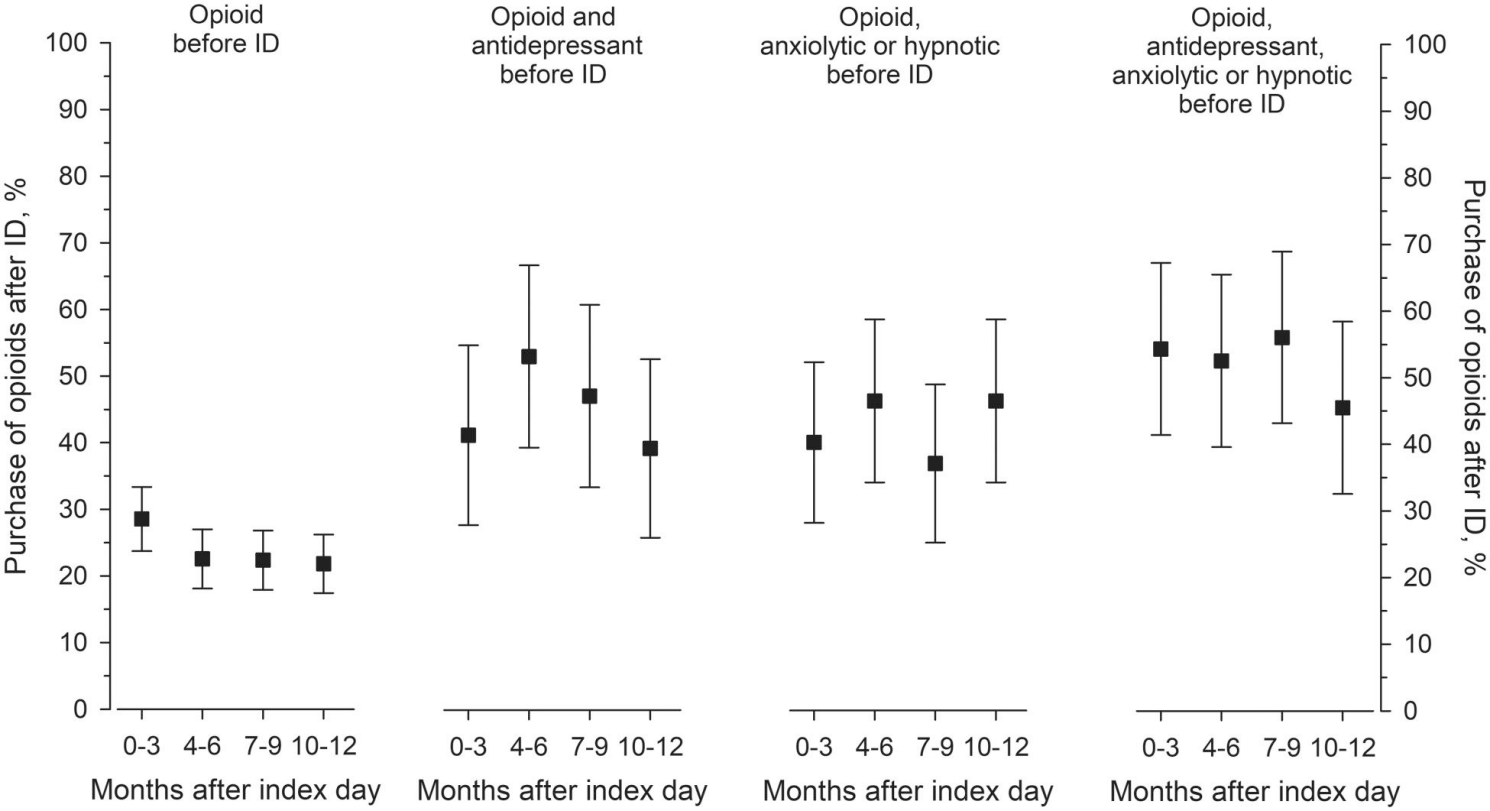
The proportion of PsA patients purchasing opioids during the year, divided into 3-month periods, after the ID. The patients are separated into four groups according to their use of opioids, antidepressants, anxiolytics or hypnotics, or the combinations of these during the year before the ID. The results are adjusted for sex and age

Opioid purchases during the year after the ID were more common among PsA patients who had purchased opioids before the ID; <10% of patients who did not purchase opioids before the ID purchased them 4–12 months after the ID compared with 35% of those who had bought opioids before the ID (Fig. 2). The likelihood of any opioid purchases after the ID or prolonged opioid use (purchase of opioids 4–12 months after the ID) increased even more if antidepressants and/or anxiolytics or hypnotics had been used in combination with opioids before the ID (Figs. 2 and 3). Of patients purchasing opioids and antidepressants as well as anxiolytics or hypnotics before the ID, 75% had prolonged opioid use (Fig. 2). Overall, 29% of cases purchasing antidepressants and 32% purchasing anxiolytics or hypnotics after the ID had prolonged opioid use compared with 14% and 16% of controls, respectively (Supplementary Table S1, available at *Rheumatology Advances in Practice* online).

In a multivariate analysis, prior purchases of opioids and drugs used in psychiatric diseases (antidepressants and anxiolytics or hypnotics) were found to increase the likelihood of prolonged opioid purchases, while sex, age and education level did not play a significant role (Table 3).

**Table 3.** Multivariable analysis to evaluate factors associated with PsA patients’ opioid purchases 4–12 months after the ID

Factors	Multivariate, OR (95% CI)	Forward stepwise, OR (95% CI)^a^
Male sex	1.03 (0.81, 1.30)	
Age (at ID)	1.01 (1.00, 1.02)	1.01 (1.00, 1.02)
Drugs purchased before the ID		
Opioids	8.35 (6.58, 10.59)	8.51 (6.71, 10.79)
DMARDs	1.23 (0.97, 1.56)	
Antidepressants	1.97 (1.44, 2.69)	1.98 (1.45, 2.70)
Anxiolytics or hypnotics	1.89 (1.39, 2.57)	1.88 (1.38, 2.55)
Education level		
Basic	1.00 (reference)	
Middle	1.11 (0.83, 1.50)	
High	0.89 (0.59, 1.34)	

OR: odds ratio; CI: confidence interval.

a Forward selection. Only those variables are shown that entered the model.

## Discussion

A great majority of the patients granted a reimbursement for PsA medication initiated DMARDs in Finland between 2010 and 2014, most often MTX. In contrast, half of them had been using DMARDs or GCs before the reimbursement decision, presumably due to their skin disease or undifferentiated arthritis symptoms. The patients were also using painkillers before the ID more often than their controls. Purchases of pain medicines decreased markedly after the initiation of DMARDs for NSAIDs and paracetamol, but not that steeply for opioids. PsA patients purchased antidepressants and hypnotics more often than their controls before and after DMARD initiation. The use of these drugs, as well as the use of anxiolytics, increased the risk of purchasing opioids even several months after the diagnosis.

According to this study, a total of 2678 PsA diagnoses were made in Finland between 2010 and 2014 in the adult population (≥17 years). This cohort is the latest part of our earlier study, in which the incidence of PsA appeared to be increasing [1]. Since then, the increase has levelled off, being now at 12.7/100 000 (L. Heimola, unpublished data). Further follow-up will show how increased diagnostic vigilance and accurate use of the classification criteria impacts the incidence in the future.

Interestingly, 51% of incident PsA patients had purchased DMARDs during the year before the PsA diagnosis, 31% of them purchasing MTX and 1% self-injected bDMARDs. These are most likely patients with difficult skin psoriasis treated with a systemic drug or ones with skin psoriasis and associated arthralgias. This phenomenon may also reflect the difficulty of setting the PsA diagnosis; if the classification criteria for PsA [13] are not met, empirical treatment trials with DMARDs may be started before making the final diagnosis and providing the medical certificate for the SR. In a Norwegian study, 18% of patients were using DMARDs 1 year before the presumed PsA diagnosis [14].

MTX was the most preferred csDMARD on the ID (71%), which is in line with the EULAR recommendations for pharmacologic treatment of PsA, [15] and the real-world register data of others [14, 16]. The Group for Research and Assessment of Psoriasis and Psoriatic Arthritis treatment recommendations for PsA places csDMARDs on the same line with bDMARDs and targeted synthetic DMARDs (tsDMARDs) when treating DMARD-naïve peripheral PsA [17], while the ACR’s 2018 guidelines for treatment for PsA recommend TNF-inhibitor biologics over csDMARDs for treatment-naïve patients in certain cases [18]. The differences between guidelines reflect both the lack of a widely agreed upon definition of severe PsA and the relatively low cost of MTX as well as its efficacy in RA [19]. In any case, in Finland the SII requires a try-out with csDMARDs before bDMARDs can be reimbursed, which explains our low figures compared with the US ones [20].

Chronic pain can be caused by several factors, including suboptimally treated inflammatory arthritis. In this trial, the PsA patients purchased more painkillers than their controls before the ID and even after it, which is in line with other studies considering pain management in inflammatory arthritis [11, 21]. For NSAIDs and paracetamol, the difference levelled off after the supposed initiation of efficient anti-rheumatic treatment, but the use of opioids remained almost at the same level. Consumption of opioids was highest during the first quarter (1–3 months) after diagnosis, when it increased to 10%. However, the peak consumption of opioids was lower than among new-onset RA and undifferentiated arthritis patients in Finland [11]. Moreover, opioid use among Finnish PsA patients is much less common than among their counterparts from the USA [21] or Denmark, where the use of DMARDs was also rather low [22]. In a US PsA study, the initiation of bDMARDs decreased the proportion of opioid users during the year after initiation [21]. Nevertheless, even after bDMARD initiation, the proportion of opioid users in that study was clearly higher than in ours after DMARD initiation (34% *vs* 10%, respectively). In our trial only a handful of PsA patients started a bDMARD during the first year, thus this effect could not be analysed. In a Finnish axial PsA study, the initiated bDMARD decreased the opioid use significantly as measured by daily doses [12]. Therefore it is possible that in Finland the initiation of bDMARDs or tsDMARDs in PsA could have a positive effect on the need for opioids, considering all the affected domains in PsA.

Since opioid treatment is not superior to non-opioid treatment in chronic musculoskeletal pain and causes more adverse medication-related symptoms [23], the fact that 8–10% of patients continued to purchase opioids after the ID, suggests that reasons other than inflammatory pain are responsible for their continued use. Fibromyalgia, chronic pain syndrome, psychiatric comorbidities and addiction to opioids may have influenced the need for continued opioid purchases [8–10].

A systematic review reported the prevalence of depression varies between 5 and 51% and anxiety between 4 and 61% in PsA patients [24]. Different criteria for defining psychiatric comorbidities may partially explain the large variance in the prevalence of these diseases [24]. Of our PsA patients, 13% received antidepressants and 13% anxiolytics or hypnotics for their mental health problems during 1 year before and 1 year after the ID. Further, data on the use of psychiatric medications by PsA patients is limited since most studies use questionnaires to evaluate psychiatric morbidity. In a Swedish study, 10.4–12.8% of the RA, PsA and AS patients were using antidepressants and benzodiazepine-related hypnotics (BRHs), which corresponds well with our results [25]. Research based on data from a large US claims database between 2009 and 2014 reported that antidepressants were used by 29.6% of PsA patients *vs* 16.6% of matched controls [26]. In our trial, 10% of the controls purchased antidepressants.

Depression and anxiety have been reported to increase disease activity and pain in PsA patients [27, 28]. In contrast, inflammation and elevated levels of pro-inflammatory cytokines have been suggested to be aetiological factors of mood disorders and depression [29]. Starting a bDMARD has been suggested to reduce the rates of depression and insomnia, as well as regular antidepressant use, in patients with PsA [30]. However, others have found that depression and anxiety may also reduce the likelihood of joint remission [9], and patients with depression seem to be more likely to discontinue their bDMARDs [31]. In the above-mentioned Swedish study, the initiation of anti-TNF or csDMARDs reduced the use of antidepressants and BRHs measured by prevalence rate ratio, yet no separate analysis of only PsA patients was published [25]. In our study, DMARD initiation did not have any effect on purchasing antidepressants, hypnotics or anxiolytics the year after the diagnosis. Also, hypnotics were more commonly used by PsA patients compared with controls and it has been reported that poor sleep associates with active joint inflammation [32]. Nevertheless, the use of hypnotics did not decrease after the ID, so presumably non-inflammatory factors played a more important role in this case.

Due to the questionable effectiveness and addictive nature of opioids, they should be prescribed with caution in inflammatory diseases. Our study may help identify patients at highest risk for prolonged opioid purchases. Of the PsA patients who had used antidepressants and anxiolytics or hypnotics the year before the ID and had also been prescribed an opioid during that year, almost 75% had prolonged opioid use. The same applied to >60% of the patients who used only an antidepressant in the preceding year and prescribed an opioid before the ID. Thus any indication of psychiatric comorbidity should warrant caution in this regard.

The controls were slightly more educated than the PsA patients, which is in accordance with others [33]. Nevertheless, there were no differences in income levels between the PsA patients and controls.

The principal strength of this study lies in its nationwide scope and availability of well-maintained public registries of high quality. Patient identification relied on diagnoses (ICD-10 codes) formulated by qualified specialists or special clinics. Therefore, we assume the PsA diagnoses are reliable, but we have no data on the fulfilment of classification criteria. In Finland, opioids are strictly available through prescriptions, thus are inclusively covered in the register we used. Additionally, the inclusion of population controls strengthens the study and allows estimation of opioid use for arthritis pain, although exact indications for analgesic therapy are not available.

A limitation of our study is the lack of clinical and behaviour data, such as disease activity, self-assessed pain scores, smoking status, alcohol consumption and body mass index. Some of the lacking data could partially explain potential failure of initiated DMARD therapy and prolonged opioid use [34]. Also, PsA patients with mild symptoms are treated in primary healthcare with NSAIDs and GC injections without applying SRs for DMARDs, thus such patients are missing from our data. Since our cases are the ones with active PsA, opioid purchases among PsA patients may be overestimated. The complex interaction between pain and mental health likely plays a role in the concomitant use of opioids and psychiatric medication in our study [35] as well as the fact that antidepressants are commonly used to treat pain. Long-term use of opioids predisposes to opioid dependence and abuse; unfortunately our data did not allow us to analyse these aspects. Paracetamol and NSAIDs can only be purchased from a pharmacy and are markedly cheaper with a prescription. But since they can also be purchased over the counter, our data regarding their use are underestimates.

Finally, we are lacking data on i.v. bDMARDs, mainly infliximab, which are given and paid by the hospitals. Nevertheless, from the data received from the Finnish ROB-FIN register (covering ≈60% of the Finnish patients receiving bDMARDs), only 62 patients were given i.v. infliximab between 2010 and 2014 as their first bDMARD with the diagnosis of PsA, and the mean date to start the first bDMARD (i.v. or self-injected) was 453 days from the PsA diagnosis. Thus we could not have missed many patients using i.v. infliximab.

In conclusion, Finnish PsA patients purchase more painkillers and antidepressants than their GP controls before their diagnosis and half of them already use DMARDs at that time. The initiation of DMARDs decreases the use of NSAIDs and paracetamol, but not that of opioids, and has no effect on the use of psychiatric medications. Purchasing opioids, antidepressants and anxiolytics or hypnotics the year before the diagnosis increases the risk of prolonged opioid use to 75%. In the future our goals will be the earliest possible diagnosis of PsA, the identification of all affected domains and a tailored initiation of early active treatment with bDMARDs or tsDMARDs if needed. This study underlines the appropriate detection and treatment of psychiatric symptoms that we know effect treatment outcomes and quality of life. When these aspects of the well-being of the patient are under control, it remains to be seen how difficult a problem the chronic pain will be. In any case, opioids should be prescribed with caution in PsA, especially in patients with psychiatric comorbidity.

## Supplementary Material

rkaf039_Supplementary_Data

## Data Availability

The data underlying this article cannot be shared publicly due to ethical and legal reasons.
