# Supplementary material for: Pain management, prolonged opioid use, initiated anti-rheumatic treatment and psychiatric morbidity in new-onset psoriatic arthritis
Source: Rheumatol Adv Pract. 2025 Apr 10;9(2):rkaf039. doi: 10.1093/rap/rkaf039 (PMC12064172; doi:10.1093/rap/rkaf039)
Supplement: rkaf039_Supplementary_Data [file rkaf039_supplementary_data.docx]

**Supplementary Table S1**. The numbers and proportions (%) of prolonged opioid users (opioid purchases 4-12 months after the ID) of psoriatic arthritis (PsA) patients and their controls purchasing antidepressants and anxiolytics or hypnotics after the ID. The age- and sex-adjusted risk ratios (RR) for drug purchases with 95% confidence intervals (CI) are shown.

|  | **Antidepressants** | |  | **Anxiolytics or hypnotics** | |  |
| --- | --- | --- | --- | --- | --- | --- |
|  | PsA patients  N=351 | Controls  N=773 | RR (95% CI) | PsA patients  N=335 | Controls  N=768 | RR (95% CI) |
| **Opioid use, N (%)** | 102 (29) | 112 (14) | 2.09 (1.66 to 2.64) | 108 (32) | 126 (16) | 1.97 (1.57 to 2.45) |
